# A Case Report of Extreme Oral Lesions: A Rare Indicator of Bullying-Associated Non-suicidal Self-Injury

**DOI:** 10.7759/cureus.44713

**Published:** 2023-09-05

**Authors:** Anabela Quitério, João Mendes Abreu, André Saura, Maria Inês Borges, Ana Corte Real

**Affiliations:** 1 Maxilofacial Surgery Department, Clinical and Academic Centre of Coimbra, Coimbra, PRT; 2 Stomatology Department, Clinical and Academic Centre of Coimbra, Coimbra, PRT; 3 Faculty of Medicine, Clinical and Academic Centre of Coimbra, Coimbra, PRT; 4 Maxilofacial Surgery and Stomatology Department, Hospital Santo António - Centro Hospitalar Universitário do Porto, Porto, PRT; 5 Forensic Dentistry Laboratory, University of Coimbra, Coimbra, PRT

**Keywords:** oral manifestations, oral ulcer, behavioral symptoms, self-injurious behavior, bullying

## Abstract

Bullying has reached epidemic proportions, affecting one in three students worldwide. A pervasive issue that carries profound physical, mental, and social consequences, significantly increasing the risk of non-suicidal self-injury (NSSI) and suicidal behaviors among those who experience this type of harassment and hazing. While physicians and most caregivers are fully aware and competent in identifying signs of self-harming behavior such as scratching, cutting, or burning the skin, oral self-injury is often overlooked as a potential indicator and is associated with unintentional soft tissue biting or specific conditions.

We present a rare case of a 14-year-old male who sought medical attention due to severe bilateral tongue ulcers, leading to his admittance to the emergency department (ED) with excruciating pain and feeding difficulties. In the reported case, although the traumatic biting of the tongue emerged as the most probable etiological factor, a specific underlying motive and contextual comprehension were initially absent. It was only after successfully establishing a foundation of trust with the patient, enabling an honest response, that it became evident that the observed lesions represented a manifestation of bullying-induced non-suicidal self-injury.

However, patients rarely openly acknowledge intentional self-inflicted lesions and/or their experiences of bullying, underscoring the necessity to maintain vigilance for alternative indicators such as behavioral changes or a noticeable decline in academic productivity.

The significance of this case also goes beyond its presentation, highlighting the largely unexplored issue of how a patient's dentofacial features can serve as substantial catalysts for bullying. Therefore, it is only through equally prioritizing awareness of uncommon signs, symptoms, and context that one can expedite early diagnosis and intervention, emphasizing the essential need for comprehensive and timely management of such cases.

## Introduction

Alarming statistics revealed that bullying has reached epidemic-like proportions. Widespread across the globe, it affects one in three students every month [1]. Encompassing traditional, sexual, and cyber forms, bullying profoundly impacts the physical, mental, and social well-being of its victims and can often go unreported by those who experience it [2]. Furthermore, individuals who experience bullying are at a significantly higher risk of engaging in non-suicidal self-injury (NSSI) as well as exhibiting suicidal behaviors [3].

NSSI is defined as intentional self-harming behavior that is not motivated by a desire to die but rather serves as a means to alleviate negative emotions. [4]. Usually appearing between the ages of 14 and 24, NSSI can serve various purposes, such as affecting regulation or self-punishment [5]. In contrast, suicide attempts often occur due to the belief that only death can provide a permanent escape from emotional pain [6].

NSSI can involve a variety of actions, including scratching, cutting, or burning the skin, typically in areas that are covered by clothing. Head-banging, hitting oneself, biting, and similar acts have also been reported, although they are less common [4,7].

Frequently overlooked as an indicator of NSSI, oral self-injury is often associated with specific disorders or organic conditions such as autism, cerebral palsy, muscular and sensory neuropathies, genetic syndromes, or even unintentional soft tissue biting [8-10]. Unsurprisingly, there are no available statistics regarding the diagnosis of oral self-injury in a dental setting [11].

This paper presents a rare case of extreme oral self-injury associated with bullying in a 14-year-old male child.

## Case presentation

A 14-year-old male (9th grade), accompanied by his parents, sought medical attention at the emergency department (ED) due to bilateral ulceration of the tongue. Additional clinical information revealed that the lesions had been present for less than a month, demonstrated a worsening progression, and were accompanied by severe pain and feeding difficulties. No additional signs or symptoms were described.

Previous medical history indicated the absence of known diseases, allergies, chronic or recent medication use, as well as any history of drug or smoking habits. Regarding the patient's dental history, the only information disclosed pertained to his ongoing orthodontic treatment, with no mention of recent or previous fillings, cavities, or parafunctional activities like bruxism or cheek and lip biting. No pertinent information was gathered concerning the family's medical history.

**Figure 1:**
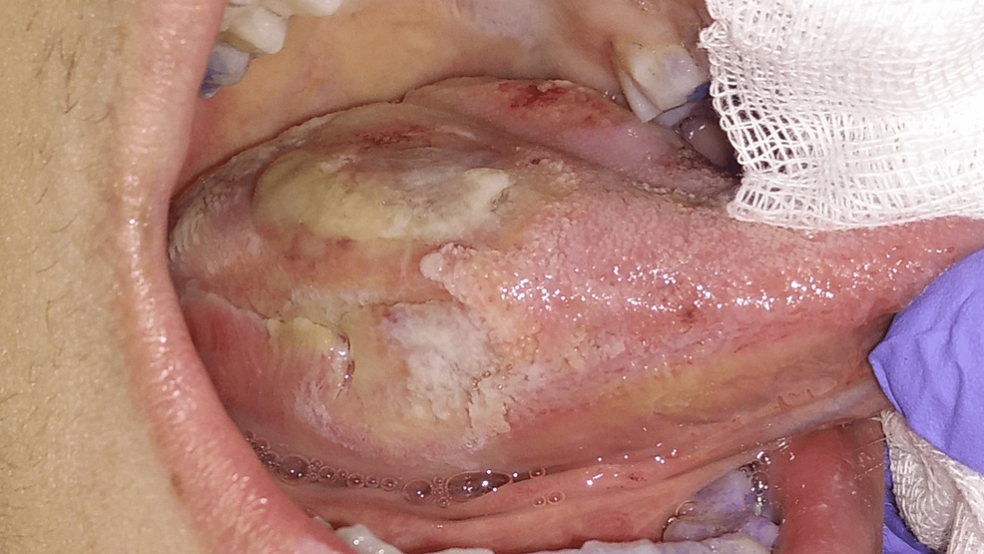
Extreme tongue ulceration (right lateral view) Extreme tongue ulceration, comprising different healing stages and covering an area of approximately 4.5 cm × 2 cm, located on the right lateral surface of the tongue.

**Figure 2:**
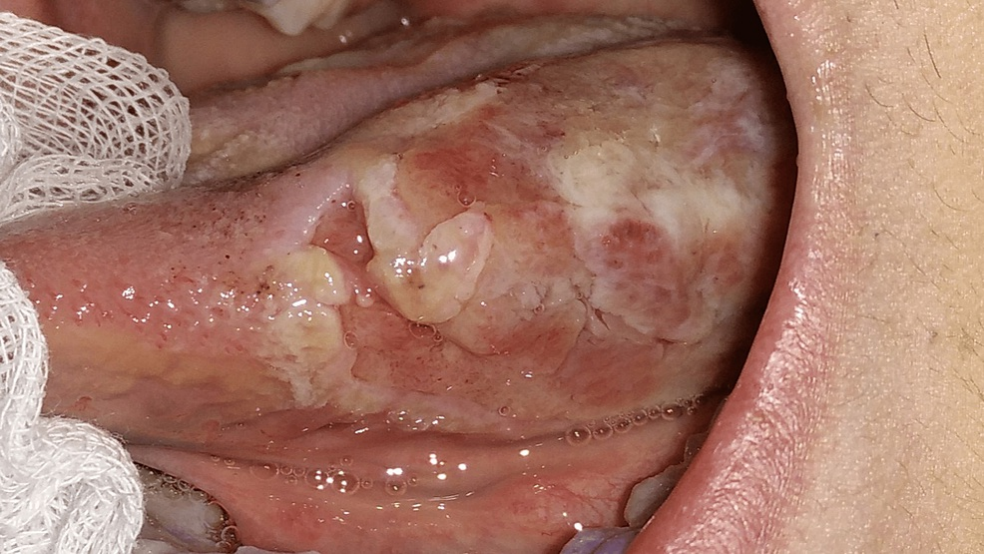
Extreme tongue ulceration (left lateral view) Extreme tongue ulceration, comprising different healing stages and covering an area of approximately 3.5 cm × 1.5 cm, located on the left lateral surface of the tongue.

**Figure 3:**
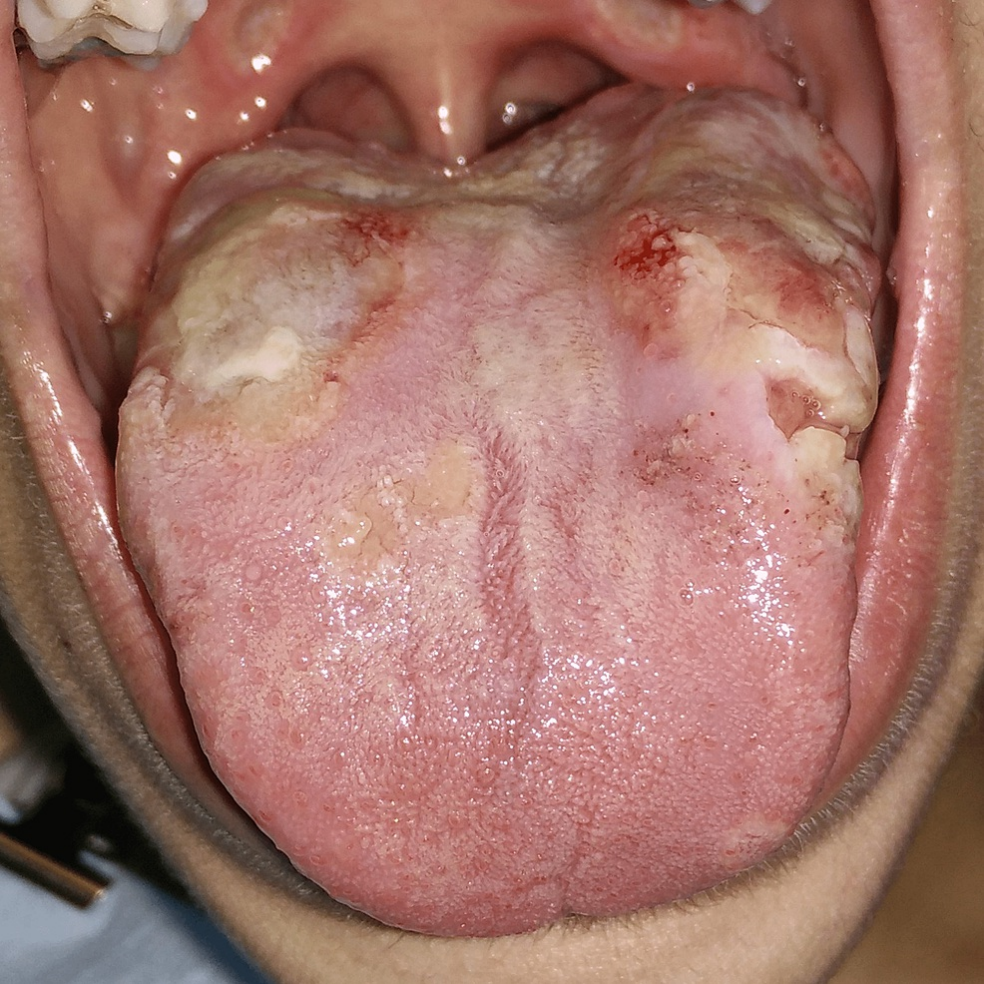
Multiple ulceration, ecchymosis and hyperkeratotic lesions of the tongue (anterior view) Extreme tongue ulceration, accompanied by multiple ecchymosis and hyperkeratotic lesions, located on the lateral and dorsal surfaces of the tongue.

Intra-oral clinical examination revealed multiple ulcers located bilaterally in the middle and posterior regions of the lateral surface of the tongue (Figures 1-2). Furthermore, a mild swelling was observed in the surrounding area, accompanied by multiple ecchymosis and hyperkeratotic lesions on the dorsal surface of the tongue (Figure 3). The tongue exhibited normal mobility and no palpable indurations. During the remaining examination, no cavities, calculus, or abnormal tooth mobility were observed. However, a slight purple staining was noticed on the vestibular surface of the teeth, attributed to the use of orthodontic rubber bands of the same color.

Extra-orally, no facial or cervical swelling, masses, or lumps were detected. The patient also denied experiencing any constitutional symptoms.

Based on the patient's clinical presentation, a suspicion of continuous traumatic biting of the tongue was raised. Permission was then obtained from the parents to conduct separate interviews with both the parents and the adolescent.

Further inquiry then revealed that the patient had recently been referred to a psychologist to address behavioral changes and a notable decline in academic productivity, which had persisted over the past 10 months. These changes, marked by persistent anger and irritability, alternating with periods of social isolation and spontaneous crying episodes, were initially observed by the parents and began shortly after the commencement of the orthodontic treatment.

Based on the patient's clinical presentation and medical history, a diagnostic hypothesis of non-suicidal self-injury involving the continuous traumatic biting of the tongue was presented.

As a result, the patient confirmed our suspicion and disclosed that a group of school peers had subjected him to persistent harassment for nearly a year, which served as a catalyst for his self-injurious behavior. The alleged reasons behind this mistreatment were his sparse facial hair and his ongoing orthodontic treatment, particularly the colored rubber bands around the brackets and the blue molar bite blocks.

Subsequently, after confirmation of the preliminary diagnosis and in compliance with the patient's request, our findings were relayed to the patient's parents, who willingly undertook the responsibility of sharing this information with the patient's psychologist, opposing the need for an internal referral for a psychiatric consultation.

The patient was then discharged and referred to the outpatient department for follow-up care, which included scheduling a low-level laser therapy session within two days. A prescription of lidocaine gel (Lidonostrum®; 1 g, five minutes before meals), betamethasone oral solution (Celestone®; 1 mL, diluted in water, three to five minutes, to gargle 4 times daily, after meals, for one week), and benzydamine mouthrinse solution (Tantum Verde®; 4 times a day, diluted in water, after oral hygiene) was also given.

Regrettably, the patient did not attend the initially scheduled and rescheduled appointments, after which telephone contact was established with the parents, who informed them that the patient would not be attending the consultation as the ulcers had started healing and the psychology consultations were still ongoing and yielding positive results. Furthermore, additional confirmation that the school was informed about the situation and that necessary measures were being implemented was also given.

## Discussion

When a patient intentionally and consciously causes harm to their own body without any suicidal intent, it is considered an NSSI. This behavior is characterized by the deliberate, direct, and calculated infliction of damage on oneself, which is socially unacceptable [12].

However, the multifactorial nature of this behavior, influenced by factors such as personality, demographics, social environment, exposure to online and media content, adverse childhood events, and neurobiological predisposition, presents challenges in identifying individuals who may be more susceptible to engaging in NSSI [13]. Nevertheless, studies have consistently revealed a strong correlation between bullying and self-harm, with the effects of exposure at an early age often persisting throughout a person's lifetime [3,14].

In the reported case, although the initial referral to the ED lacked a specific diagnostic hypothesis, several factors, including the patient's age, the timeline and location of the ulcers, the presence of lingual dorsal bruises, and the absence of systemic symptoms, raised suspicions of continuous intentional traumatic biting of the tongue [15]. A diagnosis that was later confirmed, along with the patient's admission of being a victim of constant harassment by school colleagues motivated by the patient’s physical characteristics and condition [16].

Identified as a case of oral self-injury induced by bullying, a treatment plan was developed. Due to the challenges associated with permanently impeding the self-biting of the tongue, no specific treatment option could be prescribed. Moreover, the challenges posed by the patient's ongoing orthodontic treatment and the conflicting nature of prescribing a protection device such as a mouthguard [8] further complicated the situation. Nevertheless, considering the patient's pain and the presence of oral ulcers, topical corticosteroids and anesthetics were prescribed for at-home use [17]. Furthermore, the patient was also referred for low-level laser therapy, seeking additional therapeutic benefits in managing his condition [18].

Additionally, the patient's parents, psychologist, and school, were also made aware of the situation enabling them to gain access to the necessary insights for a simultaneous intervention. This wide-ranging approach aimed to address both the detrimental effects of bullying on the patient's well-being and the pattern of self-harm, ensuring that appropriate measures could be taken to support the patient's recovery and promote his overall health [19,20].

The latter was confirmed with the indirect report of the complete healing of the ulcers, accompanied by several positive outcomes observed during the psychology sessions. Furthermore, confirmation that the school had been duly informed about the situation, and appropriate measures were implemented was also given.

## Conclusions

While it is logical to assume that dentofacial features may be a significant motivator for bullying, it is important to note that the topic remains largely unexplored. However, existing data highlights the need to treat it with the same seriousness as other promoters.

The relevance of this case lies in the atypical presentation of the NSSI, which serves as a crucial alert for healthcare professionals and caregivers. By raising awareness of uncommon signs and symptoms, one can facilitate early diagnosis and intervention, emphasizing the critical need for comprehensive and timely management of such cases.
